# Fitness for work and the aging workforce: medical assessment as a de facto mechanism of retirement in the United Kingdom after the abolition of the default retirement age

**DOI:** 10.3389/fpubh.2026.1923423

**Published:** 2026-09-03

**Authors:** Alida Langlois

**Affiliations:** Faculty of Safety Engineering, VŠB—Technical University of Ostrava, Ostrava, Czechia

**Keywords:** age discrimination, aging workforce, fitness for duty, fitness for work, mandatory retirement, occupational medicine, workplace accommodation

## Abstract

**Background:**

Fitness for work is the judgement that a person is medically able to perform a role safely. This review considers the concept to have been shaped for a younger workforce expected to leave employment at a fixed retirement age. As populations have aged and mandatory retirement ages have been abolished, it is the instrument that remains available for questions it was not designed to answer.

**Methods:**

This review traces the origins of the concept and examines what fitness for work means when applied to an aging workforce and the risks of relying on it at scale.

**Results:**

Aging is gradual and individual and is poorly suited to a categorical fitness judgement applied to a gradual process. Universal screening of older workers is impractical, and using age itself as the trigger may amount to direct age discrimination, lawful only where the employer can show that it is a proportionate means of achieving a legitimate aim. An unfit finding made without accommodation functions as an exit from work, and the limitation is not confined to older workers, since the duty to make reasonable adjustments attaches to disability alone. When the United Kingdom abolished its default retirement age in 2011, the law left capability, defined in statute by reference to health, as a route for ending an older worker’s employment. Fitness-for-work assessment may therefore function as a de facto retirement mechanism, operating where mandatory retirement once did but without its transparency or legal constraints. Where a formal unfitness procedure exists, as in France, the sequence from assessment to exit is recorded and falls more heavily on workers over 50. The United Kingdom has no equivalent procedure and no equivalent data, so the risk is structural and unmeasured.

**Conclusion:**

Fitness for work could operate as a quiet successor to the mandatory retirement that was abolished as discriminatory, not through deliberate misuse but through the unguarded working of the mechanism. Abolishing mandatory retirement was the right course. What is missing is an obligation to find accommodation, modify work, or redeploy, attached to the assessment rather than to the person assessed.

## 1 Introduction

Fitness for work is one of the oldest functions of occupational medicine. It is the evaluation of whether a worker can perform a role without undue risk to their own or others’ health and safety. A systematic review of how the fitness-for-work assessment is defined and used across occupational medicine found broad agreement on this definition and reported that the assessment is conducted primarily at recruitment and when work or health conditions change (1). The assessment supports hiring decisions, return to work after illness or injury, and continued employment in safety-critical roles. Workers were, on average, younger; the workforce left at a defined retirement age; and assessment primarily dealt with single occasions: an injury, an illness, or a specific safety-critical duty.

Each of these conditions has since changed. Workforces are aging, and participation past traditional retirement age is rising. This is reflected in data from the Organisation for Economic Co-operation and Development (OECD), where the labor force participation rate of workers aged 55–64 increased by 8 percentage points over the decade to 2018, reaching 64% on average across the OECD (2), and the employment rate of those aged 45–64 years increased by 9.3 percentage points between 2000 and 2024 (3). At the same time, many jurisdictions have removed fixed retirement ages. The United Kingdom abolished its default retirement age in October 2011, after which employers could no longer use it to compulsorily retire employees (4). Workers currently remain in employment longer, often into ages at which gradual, cumulative health change is common. Fitness for work, a concept shaped around single occasions in a younger workforce, is the instrument available when the question arises as to whether an aging worker remains able to do their job. How often it is used for that purpose is not recorded. This review asks whether the concept is fit for that purpose, and what happens when it is not.

## 2 Method

This is a narrative review. The literature was identified through searches of Scopus and PubMed, together with open searching of scholarly sources, using combinations of terms including fitness for work, fitness for duty, fitness to work, unfitness, aging workforce, older workers, health-related job loss, and capability dismissal. Searches were conducted between February and August 2026 and were updated throughout this period as further relevant material was identified. Legal material was taken from primary sources rather than secondary descriptions, using legislation.gov.uk for United Kingdom statutes, the United States Code, the Federal Register of Legislation for Australian statutes, EUR-Lex for European instruments, and published judgments for case law. Government and agency materials were obtained from the issuing body.

No formal screening protocol was applied. Results were examined against four questions. What fitness for work is and how it is defined; how capability changes with age; what the law permits when an employer ends an older worker’s employment; and whether the sequence from assessment through employer response to exit has been observed. A source was taken forward where it addressed one of those questions and either supported the argument, challenged it, or addressed the United Kingdom position. Evidence bearing against the proposition advanced in this study was sought explicitly by searching for studies linking fitness or unfitness determinations to employment outcomes in any jurisdiction and by searching for United Kingdom data on the outcomes of occupational health referral. This search located the French unfitness literature discussed in the “Fitness for Work as a De Facto Retirement Mechanism” section and located no United Kingdom equivalent.

The review is interpretive rather than systematic. No formal scoring, inclusion count, or risk-of-bias assessment was applied, and the search was not exhaustive. Searching was limited to English-language sources. Where a relevant primary source was published in another language and no English version was available, the finding is cited to the English-language source reporting it and attributed in the text accordingly.

## 3 Historical origins of the fitness-for-work concept

The practice of judging a worker’s health against the demands of the work took its modern form in the industrial era. The field is usually traced to Bernardino Ramazzini, the Italian physician whose De Morbis Artificum Diatriba, or Diseases of Workers, was first printed in 1700 and devoted each chapter to the disease associated with a particular work activity, for which he is called the father of occupational medicine (5). He visited workplaces, observed workers’ activities, and discussed their illnesses with them (5). For the next two centuries the field was concerned mainly with the injuries and diseases that work produced, rather than with assessing in advance whether a given worker could safely perform a given job.

The judgement of fitness as a gatekeeping function emerged with the factory system. Early factory legislation, such as the United Kingdom Factory Act of 1833, began to regulate who could work and under what conditions (6). That Act set its limits by age, prohibiting work in factories for those under the age of 9 (6). Because reliable registration of births did not yet exist, the age limit could not be applied without a medical judgment, and a child had to obtain a certificate from a physician or surgeon that they were of the ordinary strength and appearance of a child of the stated age (7). Teleky, surveying the history of factory hygiene, described this as the first medical pre-examination for factory work required by law (8). The Factory Act of 1844 widened the certificate, adding the words that the child was not incapacitated by disease or bodily infirmity (7).

Two mechanisms therefore appear together at the origin, and both operated at entry: A fixed age threshold, applied uniformly and without examination, and an individual medical judgment about whether a particular person was fit to be taken on. The certifying surgeons drew that distinction themselves. In a memorandum read to a Royal Commission, their association argued that the Acts concerned the preservation of health and the prevention of disease, as well as age, and that a birth certificate was not a certificate of fitness (7). Neither mechanism addressed when a worker should leave. The question raised here is what happens when a judgement developed to govern entry to work is applied at the other end of a working life. The model that formed in this period is read here as carrying the assumptions of the workforce it served. Work was physically demanding and largely manual; the hazards were discrete and visible, and the working population was young by the current standards. The interpretation that fitness assessment was shaped around a workforce expected to leave at a fixed age is offered here as a reading of the period rather than as a claim drawn from the historical literature. Built on those assumptions, fitness for work screened entrants for heavy and hazardous trades and dealt with events as they arose rather than tracking the slow capability change of workers staying in employment into later life.

A second force shaped the modern concept throughout the late 20th century, as fitness assessment became increasingly constrained by anti-discrimination and human rights law. The Americans with Disabilities Act, implemented in the United States in 1992, prohibited employment discrimination against people with disabilities and was a driving force for change in the criteria used to assess fitness for work, an effect that carried over to other countries (1, 9). In the United Kingdom the corresponding instrument was the Disability Discrimination Act 1995, and protection now sits in the Equality Act 2010 (1, 10). This pushed fitness for work toward its present form, a role-specific judgment about acceptable risk that must, in principle, rest on what the job actually requires and what the individual can actually do. The concept that an aging workforce now depends on is therefore the product of two histories, an industrial one that was built to screen discrete hazards in a young manual workforce and a legal one that bound it to individual capability and bona fide role demands. Neither history nor the question now being asked is equipped to answer whether a worker whose capability is changing gradually over years remains fit to keep a job they already hold.

## 4 Conceptual foundations and underlying assumptions

A fitness-for-work assessment compares the demands of a role against the capabilities of a person, and reaches a judgment about acceptable risk. It is, in principle, role-specific rather than person-general. The question is whether the person is fit to perform their tasks without risk to self or others (1). This framing carries assumptions that an aging workforce strains. It assumes that the demands of the role are stable, that capability can be assessed at a point in time, and that the outcome can be stated as a single verdict, with recognized categories of fit, fit with conditions or restrictions, and not fit (1, 9). The criticism developed here is directed at the assessment as a general practice of occupational medicine rather than at any single regulatory framework or at deficient local implementation. The categories themselves are not the problem. Contemporary practice already treats restricted and accommodated work as operative outcomes, holds that workplace modifications should always be considered, and treats a finding of unfitness as the exception rather than the expectation (1, 9). An accommodation-first approach is therefore not a departure from current practice. It is a statement of what current practice already recommends, and the argument is that nothing in the United Kingdom compels an employer to follow it for a worker who falls outside the statutory duty to make reasonable adjustments.

Each of these assumptions fits older workers poorly. Capability in an aging worker is not a fixed point but a moving one, and individual aging is highly variable. Health-related quality of life ranges widely within every age group, while the median quality of life varies comparatively little across age groups (11), and there are considerable individual differences in the rate at which physiological systems deteriorate (12). A point-in-time assessment captures a snapshot of a moving target. A categorical verdict also sits awkwardly with what is a gradual process. There is rarely a clear threshold at which an aging worker crosses from fit to unfit, only a widening range of conditions under which the role is or is not safe for them. The concept asks for a line where the reality offers a gradient.

## 5 Assessment considerations specific to the older worker

Applied to an older worker, fitness for work has to take into account several features that the younger-workforce model could largely ignore. The first follows from that variability. Because chronological age is not a relevant marker of healthy aging for a large segment of the older population (11), any assessment that treats age itself as the measure, rather than the individual’s actual capability, will be both inaccurate and unfair. This is not a new observation. Reviews of physical work capacity in older adults have long noted that capacity, rather than age, should be the criterion for determining whether an employee can perform a specific job (12).

The second is that capability is often modifiable through changes to the role. An aging worker who is unfit for a role as currently designed may be entirely fit for the same role with adjustment, changed hours, altered task allocation, assistive equipment, or modified pace. Workplace accommodations and job redesign can keep workers with reduced or changed capability in work, and the recognized outcomes of a fitness assessment already include fit with modifications and fit with restrictions, while workplace modifications to improve or adjust working conditions should always be considered (1, 9). A fitness assessment that judges the worker against the unmodified role, rather than against what the role could reasonably become, collapses a question about job design into a verdict about the person.

The third is that much of the age-related change is gradual and concurrent with chronic conditions. The prevalence of multimorbidity, defined as the presence of two or more of 40 morbidities, rises steeply with age. In a cross-sectional study of 1.75 million people registered with Scottish general practices, 30.4% of those aged 45–64 were multimorbid, rising to 64.9% of those aged 65–84 and 81.5% of those aged 85 and older (13). Fitness for work in an older worker is rarely about a single impairment. It is about the combined effect of several gradual changes and managed conditions on the safe performance of a role. An assessment built around discrete diagnoses can miss the cumulative picture, which often determines whether the role is safe.

## 6 Limitations of fitness-for-work assessment at scale

Even done well at the individual level, fitness for work would run into problems if relied on as a general response to an aging workforce. The first problem is practical. Comprehensive assessment of every older worker is resource-intensive and requires occupational health capacity that many employers simply lack. Provision is far from universal. Only 15% of workers worldwide have access to specialized occupational health services that provide risk prevention, health surveillance and advice to employers (14). Whatever the merits of assessment in principle, it cannot be the default mechanism for managing an entire aging workforce because the scale does not allow it.

The second pitfall concerns the trigger for assessment. If the assessment cannot be universal, some criteria must be used to select who is assessed. Assessing workers because they have reached a certain age may amount to direct discrimination, which section 13(1) of the Equality Act 2010 defines as treating a person less favorably than others because of a protected characteristic (10). Age is the one characteristic for which direct discrimination can be justified, and Section 13(2) provides that there is no discrimination where the treatment can be shown to be a proportionate means of achieving a legitimate aim (10). A trigger applied to all workers that places older workers at a particular disadvantage falls instead within the indirect discrimination test in Section 19, which carries the same justification defense (10). An age-triggered fitness regime singles out older workers for scrutiny that younger workers performing the same role do not face, and its lawfulness turns on whether the employer can justify it rather than on whether discrimination was intended. Yet a needs-based trigger, assessing only when a problem appears, returns to a reactive posture that fails to capture gradual, asymptomatic change because the problem may not appear until an incident reveals it.

Commercial aviation shows what that justification looks like where it has been made and accepted. European licensing rules restrict pilots who have attained the age of 60 to multi-pilot crews in commercial air transport, bar those who have attained 65, require medical screening every 6 months for single-pilot commercial air transport from the age of 40 (15). Age is the trigger, and it is set in regulation rather than exercised as discretion. The outcomes are also counted. Of 18 European national aviation authorities asked for data, 6 supplied eligible data of good quality, and among Class 1 pilots, those data showed a clear effect of increasing age on the medical disqualification rate, with the rate more than doubling in the 51–60 age group compared with the 41–50 group, followed by a slight decrease in the over-60 age groups, which the authors suggest might be due to a healthy worker effect caused by self-selection and medical screening, leaving the more physically and mentally fit older pilots in the workforce (15). Those who would have been found unfit have already left the population being measured. Where an age-based trigger is justified in this way, the rule is published, the assessment interval is specified, the assessment is made against a defined standard, and the results are recorded and analyzed. Outside such a regime, an employer relies on the same statutory justification without any of that apparatus, and whether it would hold is never tested because the practice is never counted.

The third pitfall is subjectivity and inconsistency. Fitness judgements about aging workers, particularly where capability is fluctuating and roles are modifiable, leave considerable room for the assessor’s judgement and, therefore, for inconsistency between assessors and the intrusion of assumptions about age. Agreement between assessors on fitness-for-work decisions can be poor. Five experienced occupational physicians independently reviewing the records of 180 applicants disagreed in 31% of cases among themselves and in 37% of cases with the examining service, and on average, 31% of applicants judged unfit by one physician had been assessed as fit by the others (16). Agreement was only marginally better where detailed medical criteria for fitness were available, which led the authors to question the validity of such judgments (16). That study concerned preemployment examination of applicants rather than the reassessment of workers already in post, but it bears on the same judgement. The same worker might be judged fit by one assessor and unfit by another, which is a poor foundation for decisions that can end a person’s working life.

A fourth limitation reaches beyond older workers. An assessment that judges a person against the role as it currently stands and that carries no obligation to consider modification bears in the same way on any worker whose capability differs from the assumed norm. Age, disability, and pregnancy and maternity are each protected characteristics under the Equality Act 2010 (10). The duty to make reasonable adjustments attaches to only one of them. A person has a disability under the Act where they have a physical or mental impairment and the impairment has a substantial and long-term adverse effect on their ability to carry out normal day-to-day activities (10). Where that definition is met, the employer should take such steps as are reasonably required to avoid the disadvantage, and a failure to comply is itself discrimination (10). Where it is not met, no such duty arises, whatever the effect on the person’s ability to do the job.

The two tests are not aligned. A fitness assessment asks whether a person can perform a particular role. The statutory definition of disability asks whether an impairment substantially affects normal day-to-day activities. A worker may be found unfit for their job and still fall outside the definition because the impairment that prevents them from working does not extend to ordinary life. For that worker, the assessment can end employment, and the adjustment duty never engages. Aging workers are only one population in that position. Workers with fluctuating conditions, workers recovering from injury, and pregnant workers may be in it too. What fails here is the model, not its application to any single group. The ageism and ableism overlap in it, because the same snapshot judgement against a role as currently designed disadvantages whoever departs from the assumed norm, whether the reason is age, impairment, or pregnancy. Workers with disabilities are subject to the same judgement, and what the employer does with it matters to whether they stay. A systematic review of 50 longitudinal studies of the employer’s role in supporting work participation of workers with disabilities found strong evidence for an association between work accommodations and both continued employment and return to work, with moderate evidence for social support and weaker evidence at the organizational level (17). The accommodation stage is therefore not only where the legal duty sits. It is where the evidence locates the difference between remaining in work and leaving it. Occupational health states its own aim in both directions. It seeks to place and maintain workers in an occupational environment adapted to their physiological and psychological capabilities and describes itself as aiming to adapt work to the workers and each worker to his or her job (1). In that regard, a critical function of the occupational health doctor is to assess whether such adaptation occurs spontaneously or whether modifications or accommodations are necessary (1). An assessment that measures a person against the role as it currently stands runs in one direction only.

## 7 Fitness for work as a de facto retirement mechanism

These limitations converge on a larger concern. When an aging worker is found unfit for the role, and no accommodation or redeployment follows, the result is that they leave work. Fitness for work has then functioned not as a tool for keeping people safely employed but as a mechanism for ending employment.

That exit through health is not hypothetical, and health-related job loss is a measurable and substantial route out of work at older ages. A study pooling seven prospective cohorts of workers who were in paid employment at least once around the age of 50 recorded just over 50,000 work exits, of which an average of 14% were health related, with the proportion ranging from 2–32% across the individual studies, and low education and low occupational grade were associated with an increased risk of health-related exit in most of them (18). In a population cohort of workers aged 50–64 years in England, health-related job loss was reported by 4.6% of men and 6.8% of women over only 2 years of follow-up, and the work-related risk factors identified were job dissatisfaction and not coping with the physical or mental demands of work (19). National figures point the same way. Among the 2.1 million people aged 50–64 in the United Kingdom who were not in work in 2023 and had left their last job at some point in the previous 8 years, 24.0% gave health reasons for leaving, an increase of 2.1 percentage points from the previous year, and a larger share than the 14.8% who gave dismissal or redundancy (20). Over the same year, the share giving retirement as the reason fell from 37.0 to 34.2% (20). These figures describe the channel rather than its misuse.

They do not show that any of these exits were engineered by an employer or driven by a fitness verdict rather than by genuine ill health, and they do not record whether any assessment took place. In the pooled cohort study, health-related exit was ascertained from the receipt of a health-related benefit or pension or from the reported reason for stopping work (18), which captures the reason given for leaving rather than the process that produced it. In the England cohort, a health-related job loss was recorded when a participant stated that a health problem was mainly or partly the reason for leaving work, and fewer than half of those reporting one said that health was mainly rather than partly the reason (19). The national figures are the reasons respondents gave for leaving their last job (20). Each of these measures records why a person says they left, not what preceded the departure. What they establish is that exit through health and capability is a well-travelled path, which is precisely why it matters whether the assessment that governs it is oriented toward accommodation or toward exit.

Where the assessment is a formal procedure, the sequence can be observed. In France, unfitness, i.e., inaptitude, is the recognition by the occupational physician that a worker’s health status is no longer compatible with their current job, and the physician reports one of three conditions to the employer: fit, fit subject to work modification, or unfit for the job (21). An unfit finding may lead to reassignment to a suitable job in the same company or to termination of the employment contract on medical grounds, and a regional study by the Ministry of Work, reported by Lesage et al., found termination to be the outcome in more than 97% of unfitness notifications (21). In a 1-year study involving 51,132 workers followed by 17 occupational physicians, the incidence of being found unfit to return to one’s job was 7.8 per thousand, and workers over 50 were more frequently found unfit, with an adjusted odds ratio of 2.63 (21). The mechanism is therefore not hypothetical, as a named procedure exists. It is recorded, it ends in exit in most cases, and it falls more heavily on older workers. The United Kingdom has no equivalent statutory procedure, no reporting requirement attached to a fitness opinion, and correspondingly no equivalent data. The risk described in this review is structural and unmeasured rather than absent, and the absence of United Kingdom evidence reflects the absence of a mechanism for recording it.

This matters because of what has been removed from around it. In the United Kingdom, compulsory retirement is a form of direct age discrimination, lawful only where an employer can show that it is a proportionate means of achieving a legitimate aim (4). Retirement and fitness were already closely tied. Reviewing the European case law in Seldon, the Supreme Court set out the legitimate aims that have been recognized in the context of direct age discrimination claims. Among them are avoiding the need to dismiss employees on the grounds that they are no longer capable of doing the job, which may be humiliating for the employee concerned, and avoiding disputes about an employee’s fitness for work at a certain age (22). A fixed retirement age was, in part, a way to avoid assessing older workers on a case-by-case basis. When that blanket mechanism was removed, the individual fitness questions it had previously allowed employers to sidestep remained.

The legal structure that replaced fixed retirement makes the substitution concrete. In the United Kingdom, once the default retirement age was repealed in 2011, retirement ceased to be a fair reason for dismissal, and an employer wishing to end an older worker’s employment had to rely on one of the remaining fair reasons, including capability (23, 24). Capability is defined in statute by reference to skill, aptitude, health, or any other physical or mental quality (23). Fairness does not follow from the reason alone. Section 98(4) makes the determination turn on whether, in the circumstances and having regard to the size and administrative resources of the employer, the employer acted reasonably in treating capability as a sufficient reason for dismissal and requires the question to be decided in accordance with equity and the substantial merits of the case (23).

A capability dismissal on health grounds therefore asks a question about health and capability of the same kind that a fitness-for-work assessment asks. The two are not the same act. A fitness opinion informs an employer, and the employer decides. Occupational medicine is explicit on this point, holding that it is not the doctor who decides which risks are acceptable but the employer with the doctor’s advice, describing the doctor’s role in the final decision as advisory (1, 9).

**Figure 1:**
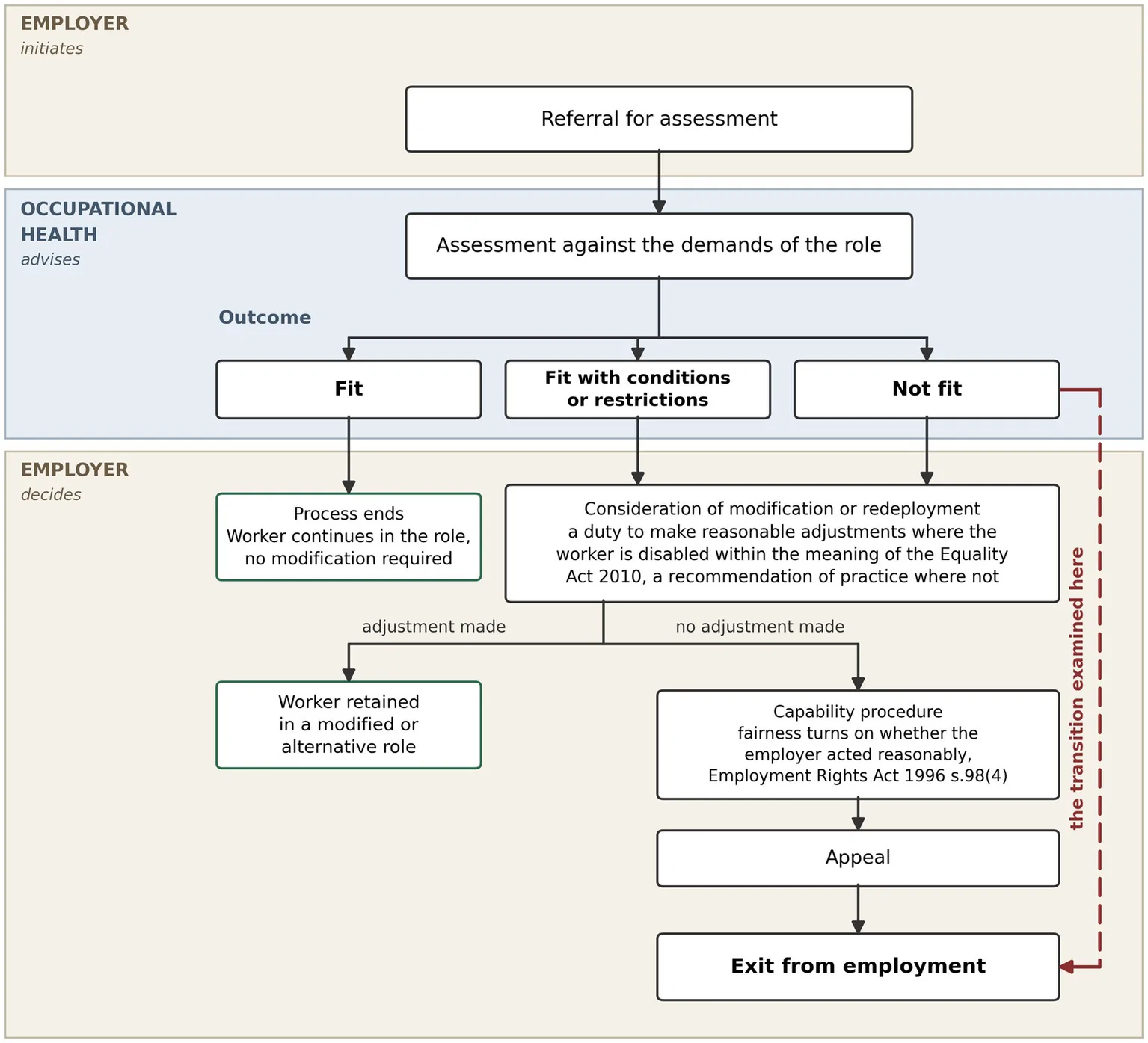
The decision pathway from referral to exit. The employer initiates the referral and makes every decision based on the clinical opinion. A fit outcome ends the process. The other two outcomes pass to the employer, where modification or redeployment should be considered before any capability procedure. A fitness opinion advises an employer and does not, in itself, complete these stages. The transition examined in this review is the one from an unfit finding to exit, in which modification and redeployment have not been considered.

Between the clinical opinion and any exit sit several distinct stages. A referral is made and an assessment carried out. The assessment produces an outcome of fit, fit with conditions or restrictions, or not fit (1, 9). Where restrictions are identified, modification of the role or redeployment should be considered, and where the worker is disabled within the meaning of the Equality Act, the employer is under a duty to take reasonable steps to avoid the disadvantage (10). If restrictions cannot be accommodated, the employer’s capability procedure may then engage, subject to the reasonableness and fair-process requirements of section 98(4) (23), with a right of appeal in case of disagreement (1). Exit is the last of these stages and the only one at which employment actually ends. Figure 1 sets out the pathway and the point at which the clinical opinion gives way to the employer’s decision. The transition this review concerns is the narrow one: from an unfit finding to exit, where modification and redeployment have not been considered. That is the point at which the assessment stops being a tool for keeping people in work. The government that removed the default retirement age agreed at the time that there was likely to be a limited increase in the use of capability procedures, and respondents to its consultation raised the prospect of capability and performance procedures being used to dismiss underperforming older workers (24). In the same response, the government acknowledged the risk that age discrimination claims would arise where performance measures are taken against an individual who, under the default retirement age, would have been retired (24). The route out of employment did not disappear when fixed retirement did. It was redirected to individual capability, and, in law, capability includes health.

The concern this raises is one of potential rather than proven practice. There is no evidence from the United Kingdom that employers are deliberately using fitness assessments to remove older workers, and this review makes no such claim. The concern is structural. The machinery currently exists for a capability or fitness finding to end an older worker’s employment, and occupational medicine treats a finding of unfitness as a last resort, to be reached only after workplace modification and enabling options, such as progressive return to work, temporary reduction of duties or changes in function or schedule, have been considered (1). Because capability is poorly predicted by age, the tool is also inherently discretionary. This is risky because the assessment relies on judgement and is applied to a group whose capability varies far more than their age suggests. It can push workers out even when no one sets out to misuse it, simply because the assessment can result in termination.

What holds this risk in check is narrower than it first appears. In the United Kingdom, the duty to make reasonable adjustments arises under section 20 of the Equality Act 2010, which is framed around a disabled person placed at a substantial disadvantage by a provision, criterion, or practice, and requires the employer to take such steps as it is reasonable to have to take to avoid that disadvantage (10). Two limits follow. The duty attaches to workers who meet the statutory definition of disability rather than to older workers as a group, and it extends only to reasonable steps. Where the duty does apply, failing to comply with it is itself discrimination against the disabled person under Section 21(2) (10). An older worker whose capability has changed gradually and who is not disabled within the meaning of the act, falls outside both the duty and that consequence. For that worker, an accommodation-first response rests on occupational health practice and on the employer’s own procedures rather than on a statutory duty.

Where a fixed retirement age was transparent, predictable, and uniform, an unfit finding is individualized, medicalized, and case-by-case. It does not announce itself as a policy of removing older workers. It presents as a neutral clinical judgement about one person’s capacity. However, if such findings fall disproportionately on older workers, are triggered by age, and lead to exit rather than accommodation, the aggregate effect can resemble the very thing that abolishing mandatory retirement was meant to end, achieved through a harder-to-see and harder-to-challenge route. Researchers examining the weak link between chronological age and actual health have already argued that numerous forced-retirement policies exist for occupations in the private sector, many of them age-based, and that their analysis suggests those policies may be outdated (11). A medicalized, individualized exit can sidestep the legal protections that a blanket age rule would trigger, while producing a similar outcome.

This is not an argument that fitness-for-work assessment is illegitimate, nor that older workers should never be found unfit for safety-critical roles. Genuine fitness concerns are real, and safety-critical work requires them. It is an argument that the boundary between protecting safety and managing older workers out of employment is easily blurred when an unfit verdict carries no obligation to accommodate and that the removal of mandatory retirement has quietly raised the stakes of where that boundary falls.

## 8 Implications for assessment practice and policy

Three conditions would have to hold for fitness-for-work assessments to support continued employment rather than end it. Assessment would have to judge capability against the role as it could reasonably be modified rather than as it currently stands therefore, the first response to a fitness concern is to identify the adjustment that makes the role safe. This accommodation-first approach is consistent with the evidence on job redesign and with the duties already imposed by disability-discrimination law (1, 9). Triggers for assessment would have to rest on role demands and observed need rather than on age. Furthermore, an unfit finding would have to carry a genuine obligation to consider redeployment or modification before it becomes an exit. These conditions reorient the assessment without removing its safety function. Two of the three already sit in occupational health practice. Workplace modification should always be considered, and a finding of unfitness is treated as the exception (1, 9). What is absent is any general requirement that they be followed. In the United Kingdom, the obligation attaches through the duty to make reasonable adjustments, which arises where the worker is disabled within the meaning of the Equality Act 2010 and extends only to the steps that are reasonable (10). A worker whose capability has changed but who falls outside that definition is owed the practice but not the duty. The policy conclusion follows from that. Abolishing mandatory retirement was the right course, and nothing here argues for its return. What the abolition left unaddressed is the absence of any general obligation to find accommodation, modify work, or redeploy before an assessment becomes an exit. That obligation should attach to the assessment itself rather than to the characteristics of the person being assessed. Where it attaches to the person, the same clinical finding carries different consequences for an aging worker, a disabled worker, and a pregnant worker, and the assessment that generated it is indifferent to which of them is in front of it.

Aviation offers a worked example of the same choice made deliberately. Considering whether the age limits for commercial pilots could be raised, the review commissioned by the European Union Aviation Safety Agency recommended extending the limit for single-pilot commercial operations from the age of 60 to the age of 65, provided that additional measures were taken, and specified them: a 6-month medical screening together with a proficiency or simulator check every 6 months between those ages (15). For multi-pilot operations, it recommended keeping the limit at the age of 65, on the grounds that allowing pilots older than the age of 65 would require additional risk-mitigation measures, such as specific tests to support aeromedical fitness decisions on an individual basis (15). The age rule was relaxed where the assessment replacing it could be specified and retained where it could not. The United Kingdom removed its default retirement age without specifying anything, and capability absorbed the difference by default.

## 9 Conclusion

This review has read fitness for work as a concept shaped for a younger workforce that left at a fixed age. Applied to an aging workforce in which mandatory retirement has been removed, it is being asked to achieve a goal for which it was not designed, and the way it answers can determine whether older workers stay in work or leave it. This is where the fitness for work model starts to fail, as aging is gradual, individual, and modifiable, while the fitness assessment asks for a point-in-time verdict. The failure is not confined to older workers, since the same judgement bears on anyone whose capability departs from the assumed norm, and the duty to accommodate reaches only some of them. It cannot be applied to everyone, and an age-based trigger for its selective application is treated in law as direct discrimination unless the employer can justify it. Most consequentially, an unfit finding, which carries no obligation to accommodate, functions as an exit from work. The legal record shows that removing fixed retirement in the United Kingdom left capability, defined in statute to include health, as a lawful route for ending an older worker’s employment. Fitness for work, therefore, has the potential to serve as a quiet successor to mandatory retirement, which was abolished as discriminatory.

It does so not through deliberate misuse but through the unguarded working of the mechanism itself. Whether it supports the aging workforce or manages it out depends on a single design choice, whether an unfit verdict is the start of accommodation or the end of employment.
